# Floating Wetland Treatment of Acid Mine Drainage using Eichhornia crassipes (Water Hyacinth)

**DOI:** 10.5696/2156-9614-8.17.14

**Published:** 2018-03-12

**Authors:** Chandimal Randunu Palihakkara, Sandun Dassanayake, Chulantha Jayawardena, Indishe Prabath Senanayake

**Affiliations:** 1 Department of Earth Resources Engineering, University of Moratuwa, Moratuwa, Sri Lanka; 2 School of Engineering, Northshore College of Business and Technology, Colombo, Sri Lanka; 3 School of Engineering, Monash University Malaysia, Bandar Sunway, Selangor, Malaysia; 4 Faculty of Engineering and Built Environment, The University of Newcastle, Callaghan, NSW, Australia

**Keywords:** acid mine drainage, floating wetland, *Eichhornia crassipes*, water hyacinth, cadmium, copper

## Abstract

**Background.:**

Acid mine drainage (AMD) is a major environmental impact associated with the mining industry. Elevated acidic conditions resulting from the discharge of AMD into the surrounding environment can cause heavy metals to dissolve and transport through water streams and accumulate in the aquatic environment, posing a risk to the health of living organisms. There have been several novel approaches in the remediation of AMD involving passive treatment techniques. The constructed treatment wetland approach is a passive remediation option that has proven to be a cost effective and long-lasting solution in abating toxic pollutant concentrations.

**Objectives.:**

The present study investigates the applicability of water hyacinth (Eichhornia crassipes), a tropical aquatic plant with reported heavy metal hyper-accumulation in microcosm floating wetland treatment systems designed to remediate AMD with copper (Cu) and cadmium (Cd) concentrations exceeding threshold limits.

**Methods.:**

Twelve water hyacinth samples were prepared with varying concentrations of Cu (1 mg/L, 2 mg/L, 4 mg/L) and Cd (0.005 mg/L, 0.01 mg/L, 0.02 mg/L). Water samples of 5 ml each were collected from each sample at 24-hour intervals for analysis with an atomic absorption spectrometer.

**Results.:**

Plant growth varied according to Cu and Cd concentrations and no plants survived for more than 14 days. There was a significant discrepancy in the rate at which the Cd concentrations abated. The rate of reduction was rapid for higher concentrations and after 24 hours a substantial reduction was achieved. There was a reduction in Cu concentration after the first 24-hour period, and after the next 24-hour period the concentrations were again elevated in the samples at initial concentrations of 2 mg/L and A4 mg/L. 4 mg/L Cu concentration was shown to be toxic to the plants, as they had low accumulations and rapid dying was evident.

**Conclusions.:**

Water hyacinth has the capability to reduce both Cu and Cd concentrations, except at an initial concentration of 4 mg/L of Cu, which was toxic to the plants.

**Competing Interests.:**

The authors declare no competing financial interests.

## Introduction

Mine wastes make up one of the largest volumes of waste materials globally, and the generation of acid mine drainage poses a major environmental threat.^1^,^2^ The high acidity in mine drainage causes the dissolution of heavy metals in the surrounding area. Elevated concentrations of heavy metals pose a substantive threat to the environment, especially when the allowable threshold limits of heavy metals are exceeded.^1^,^3^

Johnson and Hallberg reported that oxidation of iron pyrite is the primary cause of acid mine drainage (AMD) generation.^3^ Furthermore, Akcil and Koldas stated that the primary factors determining the rate of acid generation are pH, temperature, oxygen content of the gas phase, oxygen concentration in the water phase, degree of water saturation, exposed metal sulfide surface area, chemical activation energy required to initiate acid generation, and bacterial activity.^2^ Several studies have also shown that in addition to iron sulfides, other metal sulfide minerals also produce AMD and associated metal contamination depends on the type and amount of sulfide mineral oxidized, as well as the type of gangue minerals present in the rock.^2–5^ Mine drainage is largely comprised of copper (Cu), cadmium (Cd), zinc (Zn) and arsenic (As). 2–5

**Abbreviations**
*AMD*: Acid mine drainage

The present study assesses the feasibility of a floating treatment wetland application for abatement of Cu and Cd concentrations in mine drainage^6^ The applicability of floating wetland treatment was examined using a microcosm study design. Several researchers have reported that this approach can be used to comprehensively understand the natural phenomenon under study.^7–9^ Eichhornia crassipes (water hyacinth), a common aquatic weed, was used as the phytoremediation media, as this plant has been found to bioaccumulate heavy metals, and Cu and Cd in particular.^10–13^

## Methods

**Figure 1:**
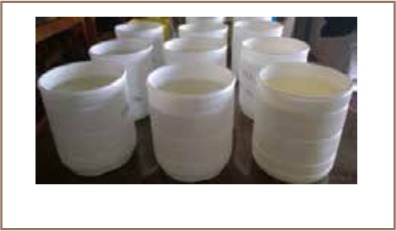
Microcosm samples containing heavy metals (copper and cadmium)

Using plastic containers (diameter: 16 cm, height: 25 cm), 9 microcosm samples were prepared, including 3 control samples, with varying concentrations of Cu^2+^ and Cd^2+^ *(Figure 1).* The sample volumes were 3.5 L each. Deionized water was used without any added nutrients, as water hyacinth grown under nutrient-poor conditions is thought to be ideal for removing heavy metals from an aqueous environment.^14^,^15^ Since AMD contains high concentrations of ferrous (Fe^2+^) each microcosm sample was initially fed with 531.615 mg of ferrous sulfate heptahydrate (FeSO_4_.7H_2_O) (initial iron (Fe) concentration was 30.5 mg/L). Apart from this Fe dissolution, the microcosm samples were not fed any heavy metals during the experiment to ensure that significant concentration reductions/increases only resulted from two effects: precipitation or phytoremediation.

To achieve the specified concentrations *(Tables 1 and 2)* Cu^2+^ concentrations, copper(II) sulfate pentahydrate (CuSO_4_·5H_2_O) were dissolved in deionized water and Cd^2+^ samples were prepared using a 1000 ppm standard Cd^2+^ solution.^16^ Initial pH values of the samples were pH 7, indicating a neutral environment. Concentrations were identified based on various case studies and prominently recorded high concentrations were used as the ceiling values for Cu^2+^ and Cd^2+^.^17–20^

**Table 1:** Copper Concentrations Prepared Using Copper Sulfate Pentahydrate

**Required concentration (mg/L)**	**Mass (mg) added to the containers**	**CuSO_4_·5H_2_O mass (mg) added**
1	3.5	13.752
2	7.0	27.503
4	14.0	55.006

**Table 2:** Cadmium Concentrations Prepared Using 1000 PPM Standard Solution

**Required concentration (mg/L)**	**Mass (mg) added to the containers**	**Volume of 1000 ppm standard solution (ml) added**
0.005	0.0175	17.5
0.01	0.035	35
0.02	0.07	70

**Figure 2:**
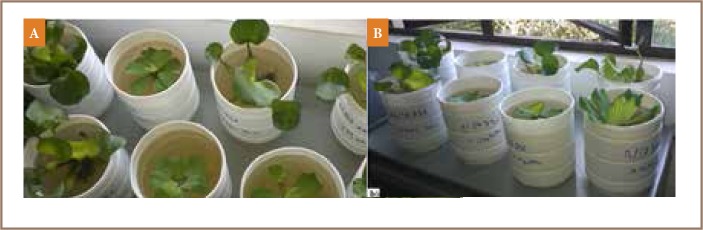
Water hyacinth planted in microcosm samples: a) plan view of the leaves; b) samples kept on the location

Samples of adult water hyacinth were obtained from Bolgoda Lake and kept in nutrient rich water in which concentrations of Cu and Cd were zero. After two weeks of acclimatization in the laboratory environment, the weight of each water hyacinth plant was measured and the total accumulated weight of the selected plants was divided equally among the number of containers. Combinations of one or more plants were used to achieve the required amount of plant weight in each sample, with an accuracy of ± 5 grams. The roots were cut to about 15 cm (as described by Muramoto and Oki) before the plants were introduced into the microcosm samples *(Figure 2).*^21^

Water samples (5 ml) were collected from each sample at 24-hour intervals for analysis. An atomic absorption spectrometer (SOLAAR) was used for the analysis of Cu and Cd.

## Results

**Figure 3:**
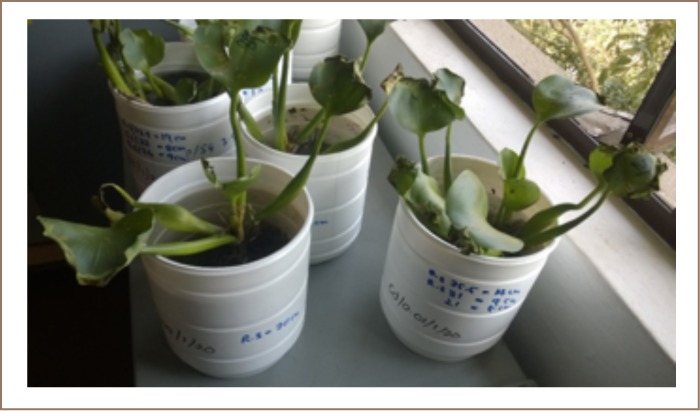
Water hyacinth plants in microcosm samples after seven days

The plants in the Cd-concentrated samples survived for 10 days, while the plants in Cu concentrations showed signs of intoxication beginning several days after the start of the experiment, but survived for a longer period. However, after 14 days, the plants at every concentration died. Figure 3 shows the appearance of plants qualitatively showing signs of intoxication 7 days after the beginning of the experiment.

Plant growth was also affected by Cu and Cd concentrations, indicating suppressed development of new roots and reduced relative growth rates.^22^

**Figure 4:**
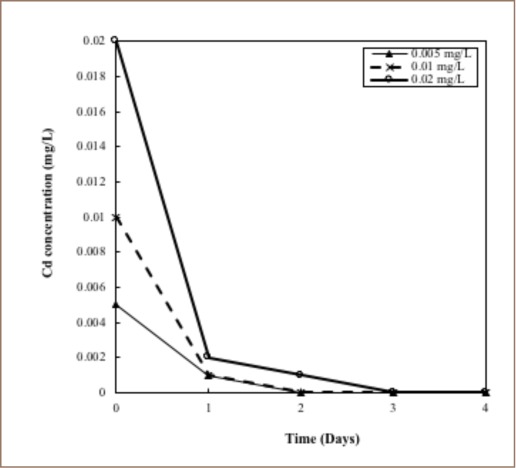
Variations of cadmium concentrations present in the water samples with respect to time

There was a significant discrepancy in the rate at which the Cd concentrations abated; the rate of reduction was rapid for higher concentrations, and after 24 hours, a substantial reduction was achieved *(Figure 4).*

**Figure 5:**
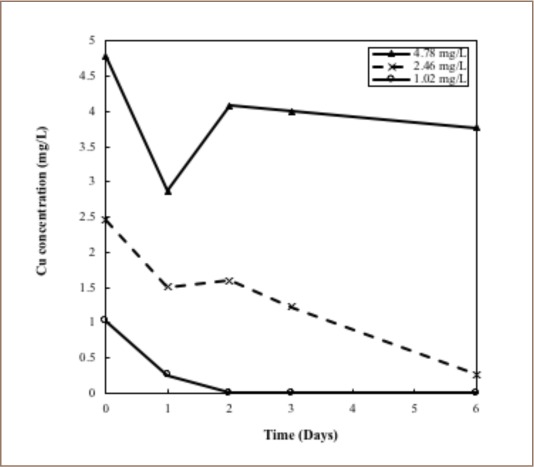
Variations of copper concentrations present in the water samples with respect to time

Concentrations of Cu showed ambiguous variations *(Figure 5).* There was a reduction after the first 24-hour period. After the next 24-hour period, the concentrations were again elevated in the samples with initial concentrations of 2 mg/L and 4 mg/L. A 4 mg/L concentration of Cu was proven to be toxic to the plants. The plants showed low accumulations and rapid dying was evident.

## Discussion

Since mineralogy and other factors causing AMD generation differ significantly across sites, each mine is unique in terms of its AMD and it was difficult to determine specific concentrations of heavy metals.^2^ Nevertheless, AMD concentration levels were selected and used in the present study according to data from previous case studies.^17–20^,^25^

During the preliminary stage of this study, experiments were conducted to determine tolerable pH values for water hyacinth and those results suggested the use of a neutral pH. In addition, according to previous studies, constructed wetlands created at active or abandoned mine sites typically receive near-neutral water. Therefore, highly acidic (pH< 6), heavy metal-contaminated AMDs were treated with primary treatment methods associated with limestone-based treatment processes.^26^

However, if the AMD concentration levels are higher than the toxic tolerance of water hyacinth and pH is low for any specific case, water hyacinth plants are not able to survive. We therefore suggest applying this floating wetland approach with water hyacinth as a secondary treatment method or in conditions further downstream where the pH is near neutral and heavy metal concentrations are below the toxic level.^27^,^28^

The high variability of Cu concentrations during the first two days may be a result of several factors; the equilibrium between Cu^2+^ in a dissolved state and Cu^2+^ adsorbed on the roots, redox potential differences, and variation in the bioavailability of Cu due to variations in pH. 23,24 The objective of the present study was primarily to determine the metal uptake of entire plants and to observe metal concentration reductions in AMD. Therefore, accumulation of metals in each individual part of the water hyacinth plant was not studied. However, several studies have reported that water hyacinth accumulates higher concentrations of heavy metals in the roots than in the shoots.^12^,^14^,^29^

Since the study's primary concern was AMD discharge associated with fresh water systems, the effect of salinity was not studied. However, no relationship between salinity and heavy metal removal by water hyacinth was found in the available literature.

Because water hyacinth is a floating aquatic plant, there is no need for a floating treatment bed, therefore eliminating associated design complications.^30^,^31^ A number of methods by which water hyacinth plants can be effectively used to generate bio-gas have been suggested, and therefore, rapidly growing and invasive water hyacinth plants can be used to generate energy after their use in remediating mine drainage. 6,32,33

## Conclusions

Floating wetland microcosm samples showed substantial reduction in heavy metal concentrations, indicating that water hyacinth can be used as a treatment media for FTWs. The only form of precipitation observed was a brownish sludge due to iron(III) hydroxide. Reductions in Cu and Cd concentrations should primarily be a result of the phytoremediation action of water hyacinth, as at an initial pH value of 7, no reactions between Fe^2+^and Cd^2+^ or Fe^2+^ and Cu^2+^ have been reported in the literature.

The present study demonstrates the applicability of water hyacinths in floating wetland treatment for the remediation of AMD, as long as the plants' survival is not threatened by the drainage conditions.
